# Molecular abnormalities scale across three models of cerebral injury

**DOI:** 10.3389/fnmol.2026.1844343

**Published:** 2026-08-25

**Authors:** Volha Liaudanskaya, Matthew J. Robson, Nicole L. Vike, Sumra Bari, Christopher J. O’Connell, Orlando S. Hoilett, David L. Kaplan, Thomas M. Talavage, Eric A. Nauman, Semyon Slobounov, Hans C. Breiter

**Affiliations:** 1 Department of Biomedical Engineering, University of Cincinnati, Cincinnati, OH, United States; 2 Department of Biomedical Engineering, Tufts University, Medford, MA, United States; 3 Neuroscience Graduate Program, University of Cincinnati College of Medicine, Cincinnati, OH, United States; 4 Division of Pharmaceutical Sciences, James L. Winkle College of Pharmacy, University of Cincinnati, Cincinnati, OH, United States; 5 Department of Computer Science, University of Cincinnati, Cincinnati, OH, United States; 6 Department of Mechanical Engineering, University of Cincinnati, Cincinnati, OH, United States; 7 Department of Kinesiology, Pennsylvania State University, State College, PA, United States; 8 Department of Psychiatry, University of Cincinnati College of Medicine, Cincinnati, OH, United States

**Keywords:** cortical injury, hexosamine biosynthesis pathway, metabolism, traumatic brain injury, mitochondrial dysfunction

## Abstract

Human studies have demonstrated that repetitive head acceleration events (HAEs) disrupt metabolic homeostasis, raising the question of whether similar biochemical cascades scale across diverse platforms, from human to rodent to cell, for other traumatic brain injuries (TBIs). This study analyzed molecular disruptions in (i) humans with repetitive HAEs, (ii) mice with singular blast-elicited HAE with loss of consciousness, and (iii) a human 3D *in vitro* model of blunt injury. Humans exhibited metabolic, protein glycation, and epigenetic regulation abnormalities, with 30 pre-season metabolites predicting post-season levels with 89% accuracy. *In vivo* and *in vitro* RNA sequencing studies revealed convergence in 211 genes, with 123 metabolic genes mirroring human results. Regression analysis reflected an unprecedented 86% information scaling between platforms, namely, cell culture data predicted 86% of the information in the murine data. Athlete data linked these pathways to HAEs and motor behavior, highlighting convergent molecular disruptions across TBI models in metabolic homeostasis required for neural information processing. This approach across three platforms has not been shown before for brain-related disorders.

## Introduction

Brain trauma arises from diverse external sources (e.g., direct and indirect impacts, blast waves, whiplash events), delivering momentum to the tissues within the cranial space and resulting in a broad range of symptoms and symptom severity. The inevitable heterogeneity of clinical brain trauma, combined with substantial disparities between experimental models, has provided limited insight into what might be convergent cellular and molecular mechanisms underlying the pathology and symptoms of traumatic brain injury (TBI). The presence of similar mechanisms from different injury models would provide therapeutic targets if mechanisms at one level scaled to another level (i.e., if across-model scaling occurred). The issue of scaling, or how information from one level of organization predicts information at another level of organization (Kim et al., 2010), is a fundamental challenge that needs to be addressed if science is going to bridge the gap between (a) preclinical studies about mechanism and (b) human studies with ethological validity regarding the actual clinical phenomena. Scaling appears to apply to natural systems (Freyer et al., 2012) such as the logarithmic spiral observed with the flight path of falcons (Tucker et al., 2000), the shape of conch shells, and wind in cyclones. It emerges from scale invariance and is a core feature of laws or objects that don’t change if measures of time, length, or other parameters are dilated or contracted by a common factor (Kim et al., 2010).

To date, preclinical models of TBI have demonstrated alterations in cellular and mitochondrial metabolism, suggesting some consistencies without quantitation of a convergent pathology or potential scaling (Hubbard et al., 2019; Liaudanskaya et al., 2023; Lyons et al., 2018; Yonutas et al., 2016). Changes in TCA cycle-related enzymes (Xing et al., 2012), mitochondrial metabolism (Hubbard et al., 2019; Lyons et al., 2018; Yonutas et al., 2016), fructose metabolism (Agrawal et al., 2016), and lipid peroxidation (Lamade et al., 2020) have been reported across a range of *in vivo* and *in vitro* injury models. Large gaps remain in the identification of convergent, injury-defining pathways with consistent metabolic alterations across TBI models. For instance, although studies with human, *in vivo*, and *in vitro* platforms consistently report injury-induced mitochondria dysfunction, no studies have identified common metabolic signatures between such distinct injury models via scaling across platforms (e.g., tissue culture to murine model) and within platform (e.g., from miRNA to metabolite to behavior in a human model). For these examples, scaling would mean the demonstration that molecular information with one platform predicted a majority of molecular information for a distinct platform, or that mediation/moderation relationships were observed for human clinical and molecular measures which were consistent with observations *in vivo* and *in vitro*. These comparisons are critical to the assessment of platform relevance to each other, as well as toward common pathway targets for future studies of injury mechanisms.

**FIGURE 1:**
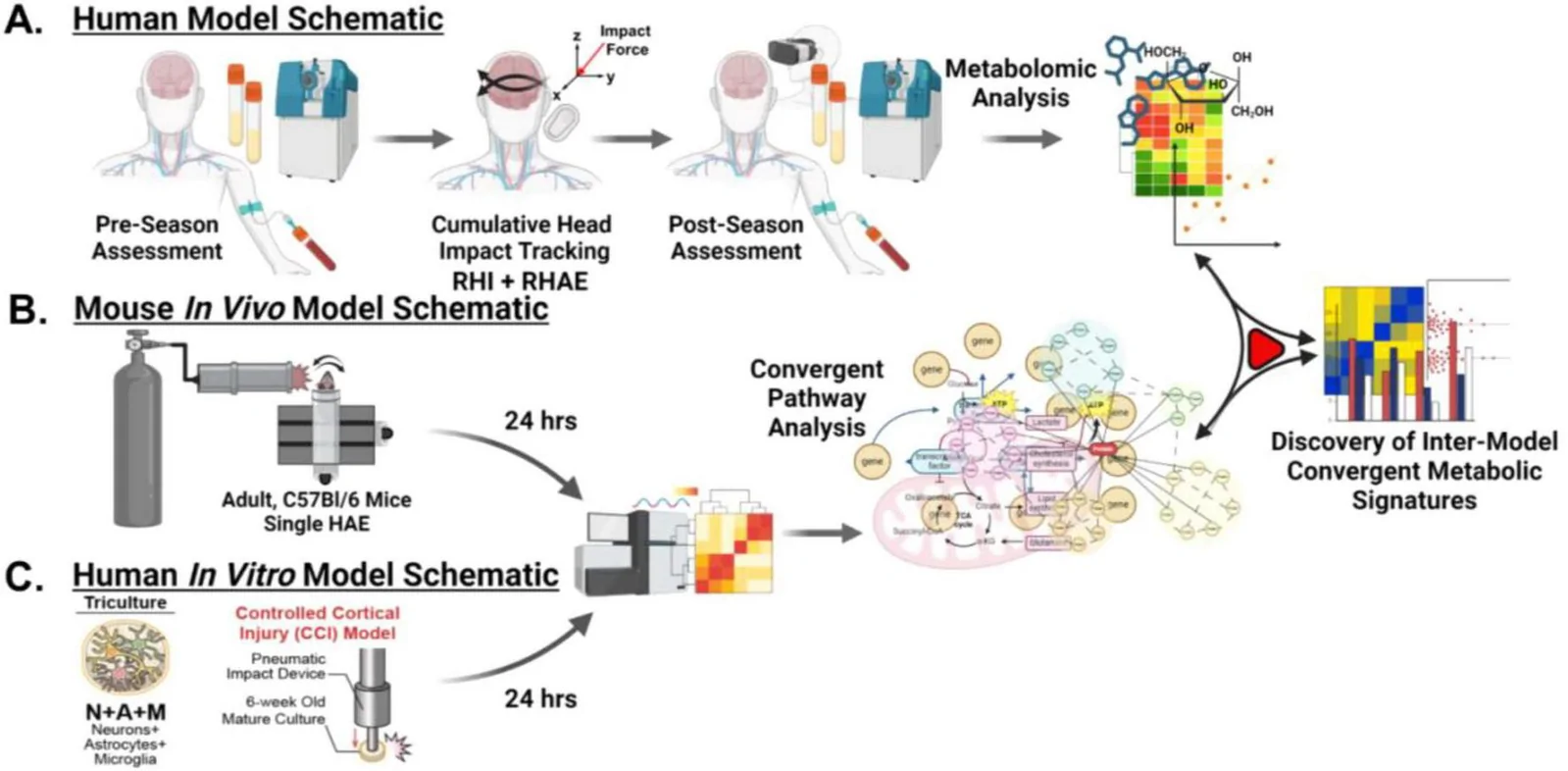
Multimodule platform to validate the human data-driven hypothesis of the injury-induced molecular alterations. Schematic depicting experimental workflow to derive convergent pathways from panel **(A)** metabolomic and virtual reality (VR) motor coordination datasets from collegiate American football athletes (*n* = 23) with documented repetitive head acceleration events (HAEs) over one athletic season, **(B)** mouse *in vivo* model of head acceleration injury 24 h after injury induction (*n* = 3 for sham and TBI; model composed of ipsilateral cortex from C57Bl/6 subjects), and **(C)** inter-model RNA sequencing data sets from the human 3D *in vitro* model of the controlled cortical impact (CCI) injury (*n* = 6 for sham and CCI; the model composed of induced neural stem cell-derived neurons, primary astrocytes, and induced pluripotent derived microglia from two healthy human donors YZ1 and ND41866*F).

The current project sought to identify statistically convergent molecular signatures across three distinct models of cerebral injury, which demonstrated scaling between platforms and within platform (Figure 1). These three distinct models differed in cellular complexity and organization, maturation, and timing of injury. These models were: (i) across-season study of collegiate-student athletes with ethologically recorded repetitive head acceleration events (HAEs), (ii) *in vivo* murine brain tissue with a multimodal injury model that included blast wave and linear acceleration/deceleration exposure, and (iii) human iPSC-derived 3D *in vitro* brain-like tissue scaffolds with a controlled cortical impact (CCI) injury model. Within the current study, we used human metabolome data to guide subsequent transcriptome and tissue-level analyses for both *in vivo* and *in vitro* platforms, using a true bedside-to-bench structure. We hypothesized that the three distinct, heterogeneous models for brain trauma share convergent cellular and mitochondrial metabolism alterations with a high level of scaling across platforms. Identification of this commonality and scaling was followed by targeted secondary analysis of human athlete data based on *in vivo* and *in vitro* findings, following a bench-to-bedside structure to link metabolic alterations to functional consequences of TBI. This work represents a re-analysis of datasets derived from two previously published *in vitro* and human studies (Liaudanskaya et al., 2023; Vike et al., 2022b) along with unpublished murine data, examined from a new transdisciplinary perspective to address questions that were not the focus of the original reports.

## Materials and methods

### Human athletes across a football season

Human datasets analyzed in the present study were originally collected as part of previously published investigations addressing distinct scientific questions (Vike et al., 2022a,b). In the current work, these datasets were re-examined under a new integrative framework focused on cross-platform molecular convergence and scaling. As such, participant enrollment, data acquisition, and primary processing procedures follow established protocols from those studies, while all downstream analyses and interpretations reported here are novel.

### Human head acceleration events (HAEs)

#### Participant recruitment and data collection

Written informed consent was obtained from each participant in accordance with the Pennsylvania State University Institutional Review Board and the Declaration of Helsinki. Twenty-three male collegiate American football athletes (mean age = 21 ± 2 years) participated during the 2015 competitive season as previously described in (Bari et al., 2021; Chen et al., 2021; Papa et al., 2019; Slobounov et al., 2017; Vike et al., 2022a,b; Supplementary Table 2). All participants were active roster members and regular starters, ensuring sustained exposure to repetitive head acceleration events (HAEs) across the season. Athletes completed study assessments at two defined time points: prior to the initiation of any contact practices (pre-season) and within 1 week following their final regular-season game (post-season).

At each time point, participants underwent virtual reality (VR)-based functional testing and venous blood collection for serum metabolomic analysis. Throughout the season, HAEs were continuously monitored during all contact practice sessions using helmet-mounted inertial sensors [BodiTrak, Head Health Network (Slobounov et al., 2017)], allowing quantification of individual head impact exposure between pre- and post-season assessments.

Participants were excluded if they had a diagnosed concussion within the 9 months preceding the pre-season assessment, a known neurological disorder, a lower-extremity injury that could interfere with balance or task performance, or if they were unable to complete the full competitive season. All athletes were enrolled in institutionally supervised training, nutrition, and lifestyle management programs for the duration of the study.

#### Virtual reality (VR) testing

Participants completed virtual reality (VR)-based functional assessments at both pre-season and post-season time points. The VR protocol is a previously validated paradigm designed to detect functional alterations in motor control and coordination associated with mild traumatic brain injury (Luria et al., 1973; Slobounov et al., 2006). All VR environments and software were developed and provided by HeadRehab, LLC (Chicago, IL), and were integrated with motion-tracking and interactive hardware components. The virtual environment consisted of a simulated “moving room” presented under two conditions: (i) global motion of the entire visual field coupled to participant movement, and (ii) selective motion of isolated visual components (e.g., lateral walls). Stimuli were displayed on a 3D television system connected to a laptop running the Sideline v8.1 software (HeadRehab, LLC), configured at a resolution of 1920 × 1080 pixels with a 16:9 aspect ratio. Head kinematics were recorded using a VCube head-tracking device, which captures translational and rotational motion via inertial sensors. Participant interaction with the virtual environment was achieved using a MoBar interface (Xbox controller), allowing navigation and task execution.

The VR protocol comprised three functional modules: balance (Bal), reaction time (RT), and spatial memory (SM). Individual module scores were normalized and subsequently combined to generate a composite performance score (Comp).

Balance module: postural stability was assessed by quantifying the ability to maintain a fixed stance during dynamic visual perturbations. Athletes were instructed to stand in a tandem Romberg position during 30-s trials (Romberg, 1853). Testing began with a baseline condition in which the visual field remained static, followed by six perturbation trials involving alterations along one of three rotational axes (yaw, pitch, or roll). Perturbations were applied using two methods: (i) anterior–posterior whole-room oscillations with a 10 cm displacement at 0.3 Hz, and (ii) lateral whole-room roll movements ranging from 10° to 30° at 0.3 Hz. Deviations from baseline posture along all three axes were recorded using a balance plate and interpolated into a performance score ranging from 0 to 10, with higher scores indicating greater postural stability.

Reaction time module: the reaction time module quantified the latency of postural correction in response to unpredictable changes in visual motion. Participants stood with feet shoulder-width apart and hands positioned on their hips while the virtual environment oscillated along the anterior-posterior axis at 0.2 Hz. At randomized intervals, the direction of motion shifted to the medial-lateral axis. Upon detection of this shift, participants were instructed to bend at the waist in the direction corresponding to the visual perturbation. Both the timing and direction of visual shifts were randomized across trials. Response latencies were recorded using accelerometers and reported in milliseconds. One practice trial preceded four experimental trials. Reaction time and anticipatory errors were interpolated and converted into a normalized score ranging from 0 to 10, with higher scores reflecting faster and more accurate responses.

Spatial memory module: spatial memory performance was evaluated using a virtual navigation task. Participants were initially presented with a randomized virtual pathway containing multiple directional turns leading to a target location, followed by a return path to the starting point (Slobounov and Sebastianelli, 2021). Path configurations were randomized between participants to minimize learning effects. After observing the full pathway, participants were instructed to reproduce the route using the handheld controller, with prior familiarization allowed. Successful navigation concluded testing. If unsuccessful, the pathway was replayed and participants were provided up to two additional attempts. Performance scores (0–10) were calculated based on the proportion of time spent on the correct path, with a penalty of 3.33 points applied per navigation error. Participants who failed all three attempts received a score of zero.

Composite score: the composite VR score (Comp) was calculated by summing the normalized scores from the balance, reaction time, and spatial memory modules and rescaling the total to a 10-point maximum.

#### Blood collection protocol

Venous blood samples (5 mL per participant per time point) were collected at both pre-season and post-season assessments for metabolomic analysis. Blood was processed to isolate serum, which was aliquoted into two barcoded tubes per sample and stored at −70 °C until shipment. Samples were transported to Metabolon, Inc., (Morrisville, NC, USA) for blinded metabolomic profiling. Details of metabolite quantification are summarized below (under Metabolite quantification) and have been previously described (Bari et al., 2021; Vike et al., 2022a,b).

#### Metabolite quantification

Serum samples were processed and analyzed by Metabolon, Inc., using standardized untargeted metabolomics workflows. Upon receipt, samples were assigned unique identifiers and stored at −80 °C until processing. Sample preparation was performed using an automated MicroLab STAR^®^ liquid handling system (Hamilton Company, Reno, NV, USA). Proteins were precipitated with methanol using a mechanical shaker (Glen Mills GenoGrinder2000), followed by centrifugation. The resulting extracts were divided into multiple fractions for parallel chromatographic analyses: two fractions for reverse-phase (RP) UPLC-MS/MS with positive ion electrospray ionization (ESI), one fraction for RP UPLC-MS/MS with negative ion ESI, one fraction for hydrophilic interaction liquid chromatography (HILIC) UPLC-MS/MS with negative ion ESI, and one fraction reserved as backup.

Samples were briefly dried using a TurboVap^®^ system (Zymark) and stored under nitrogen prior to analysis. Metabolomic profiling was performed using ultrahigh-performance liquid chromatography coupled with tandem mass spectrometry (UPLC-MS/MS) on a Waters ACQUITY UPLC system interfaced with a Thermo Scientific Q-Exactive high-resolution mass spectrometer equipped with a heated electrospray ionization (HESI-II) source and operated at a mass resolution of 35,000. Dried extracts were reconstituted in analysis-specific solvents containing internal standards to ensure chromatographic and injection consistency.

Hydrophilic metabolites were analyzed under acidic positive ion conditions using a C18 column (Waters UPLC BEH C18, 2.1 mm × 100 mm, 1.7 μm) with water-methanol gradients containing 0.05% perfluoropentanoic acid (PFPA) and 0.1% formic acid. Hydrophobic compounds were analyzed under acidic positive ion conditions using the same column with higher organic solvent content. Basic negative ion analyses were performed using a separate C18 column with methanol-water gradients containing 6.5 mM ammonium bicarbonate (pH 8). HILIC analyses were conducted using a BEH Amide column (2.1 mm × 150 mm, 1.7 μm) with water-acetonitrile gradients containing 10 mM ammonium formate (pH 10.8). Mass spectrometry acquisition alternated between full MS and data-dependent MS/MS scans using dynamic exclusion, with scan ranges spanning approximately 70–1000 m/z.

Data processing was performed using Metabolon’s proprietary bioinformatics pipeline, which integrates laboratory information management systems, peak detection and identification software, quality control tools, and data visualization platforms. Metabolites were identified by comparison to reference libraries of authenticated standards based on retention index alignment, accurate mass matching (±10 ppm), and forward/reverse MS/MS spectral similarity. This multiparametric approach enabled confident identification of more than 3,300 biochemicals.

Metabolite abundance was quantified using area-under-the-curve integration. To correct for inter-day instrument variability, data were normalized within run-day blocks by median scaling to one, followed by proportional adjustment of individual values. Normalized data were subsequently log-transformed for downstream analyses.

#### Head acceleration event (HAE) monitoring

Head acceleration exposure was monitored during all contact practice sessions throughout the competitive season (maximum of 53 sessions) using the BodiTrak helmet-mounted sensor system (Head Health Network) (Slobounov et al., 2017). Sensors were positioned on the inner surface of each helmet between the shell and padding prior to each practice. Game sessions were not monitored in accordance with NCAA guidelines and to minimize disruption. Previous work indicates that the majority of HAEs occur during practices rather than games (Lee et al., 2021).

Sensors recorded peak translational acceleration (PTA) magnitudes and impact locations in G-units. Impacts below 25 G were excluded based on evidence indicating minimal contribution to brain injury below this threshold (McCuen et al., 2015). HAEs were categorized into moderate (25–80 G) and high-magnitude (>80 G) events. For each athlete, cumulative and average PTA metrics were calculated and normalized to the number of monitored practice sessions, yielding four exposure measures: cumulative PTA between 25 and 80 G (cHAE25G), cumulative PTA > 80 G (cHAE80G), average PTA between 25 and 80 G (aHAE25G), and average PTA > 80 G (aHAE80G).

#### Statistical analyses

Random forest: these methods and subsequent results have been published previously in Vike et al. (2022b) iScience, 2021, and were used here, with the inclusion of unidentified metabolites, to guide further analyses. Random forest was used to determine which metabolites were most important to discriminate between Pre and Post-season (Breiman, 2001). Random forest modeling was applied to identify metabolites that most strongly discriminated between pre-season and post-season samples. This analytical framework has been previously described and was employed here with the inclusion of unidentified metabolites to inform downstream analyses. For each decision tree, a randomly selected subset of samples was used for model training, while the remaining out-of-bag (OOB) samples were used for internal validation. This process was iterated across thousands of trees to construct the final forest.

Model performance was assessed using OOB error rates, calculated by comparing predicted class labels with true sample classifications. Metabolite importance was quantified using mean decrease accuracy (MDA), computed by permuting individual variables (metabolites) and reassessing classification accuracy. Variables whose permutation resulted in a marked reduction in accuracy were considered highly informative. The top 30 ranked metabolites, including unidentified features labeled with an “X-” prefix, were retained for further integrative analyses.

### *In vivo* model

Adult, male wild-type C57Bl/6J (Jackson Labs Strain No.: 000664) subjects between the ages of 9–12 weeks were used for all experiments. Subjects were group-housed and maintained at the University of Cincinnati vivarium on a 14:10 light/dark cycle and given access to food/water *ad libitum*. All experimental procedures conducted during the light cycle following a 1-week facility acclimation. All procedures were conducted in accordance with the University of Cincinnati Animal Care and Use Program (ACUP), and the standards set by the Institutional Animal Care and Use Committee (IACUC) at the University of Cincinnati on animal protocol # 20-11-05-01.

#### *In vivo* HAE-elicited mild TBI

Scalable, murine model for closed head multimodal TBI resulting in exposure to a shock wave combined with an HAE was utilized for *in vivo* studies (Collins et al., 2022; Hetzer et al., 2024; Logsdon et al., 2020; O’Connell et al., 2022, 2024; Reeder et al., 2022; Turner et al., 2013). Multimodal model for mTBI was developed using scaling parameters for murine subjects and results in a rapid acceleration-deceleration of the cranium in the absence of a direct skull impact. Subjects were acclimated to the testing facility for a 30-min period prior to injury or sham treatments. All subjects were anesthetized with 4% volume-to-volume isoflurane (VetEquip). Subjects were induced (Plane III, paralysis of intercostal muscles) and immediately subjected to mTBI or sham treatments as previously described (Logsdon et al., 2020). Subjects were placed within a polyvinylchloride (PVC) shield, perpendicular to waveform, shielding internal organs from injury, but leaving the head freely available to move. Device consists of a 2-piece machined steel shock tube apparatus, driven by compressed helium gas (Airgas). The driver and driven sections of the apparatus compress a 0.003′′ Mylar^®^ membrane (ePlastics). Shock waves generated were scaled in intensity and total duration for murine subjects (Supplementary Figure 1; Reeder et al., 2022; Turner et al., 2013). Sham subjects were exposed to anesthesia, shielding tube, and noise generated, without exposure to waveform or cranial acceleration/deceleration. Immediately following mTBI or sham treatments, subjects were immediately removed, and time to regain righting reflex (RRT) was recorded prior to subjects being returned to home cages (Supplementary Figure 2).

#### Tissue collection

Twenty-four hours post-injury, subjects were sacrificed by rapid decapitation, and cortical tissue samples, ipsilateral to the waveform were collected. Samples were isolated and collected in RNA-later for processing prior to undergoing RNA sequencing (RNA Seq) analysis.

#### RNA-sequencing

RNA isolation and directional RNA sequencing (RNA-seq) were performed by the GESC at the University of Cincinnati. RNA was isolated (*N* = 3/group) utilizing the *mir*VanaTM miRNA Isolation Kit, with Phenol (ThermoFisher Scientific). RNA quality was determined using a Bioanalyzer (Agilent, Santa Clara, CA). All RNA samples for sequencing purposes displayed a 260/280 ratio of between 1.94 and 2.01 (obtained via analysis using a NanoDrop Microvolume Spectrophotometer, ThermoFisher) and passed all quality control checks using an Agilent Bioanalyzer available within the University of Cincinnati Genomics, Epigenomics and Sequencing (GESC) Core facility. To isolate the polyA RNA, NEBNext Poly(A) mRNA Magnetic Isolation Module (New England BioLabs, Ipswich, MA) was used, and 1 μg of high-quality total RNA was utilized for sequencing. The NEBNext Ultra II Directional RNA Library Prep Kit (New England BioLabs), a dUTP-based stranded library, was utilized for library preparation. After library Bioanalyzer QC analysis and quantification, individually indexed and compatible libraries were proportionally pooled and sequenced using the Nextseq 550 sequencer (Illumina, San Diego, CA). Approximately 25 million pass filter reads per sample were generated. In order to identify differentially expressed genes, bioinformatic analysis was conducted using Illumina BaseSpace by the University of Cincinnati GESC. RNA-seq Alignment App version 2.01 (Illumina) was utilized to create BAM files and data QC report. BAM files were then introduced to RNA-Seq Differential Expression App version 1.01 (Illumina).

#### Genomic data processing

The differential expression data of compiled reference genes, merged gene counts, and DESeq2 data were reviewed and downloaded from the online Illumina BaseSpace platform for supplemental data analysis. Supplemental data analysis was conducted utilizing the R/Shiny-based Interactive Gene Expression Analysis Kit (iGEAK), which aggregates gene annotations, sample-group information, and raw count matrices from BaseSpace-derived datasets. Principal component analysis (PCA) plots and a Pearson correlation plot based on transcriptomes were generated and reviewed before proceeding with further analysis.

#### Gene ontology enrichment analysis

Transcriptional regulators that may modulate expression of produced gene sets within RNA-sequencing studies were identified using EnrichR (Kuleshov et al., 2016). Gene ontology enrichment analysis (GOEA) was performed using select DEG lists to determine over/underrepresentation of biological processes using the *Mus musculus* gene ontology set. Biological processes were identified using a Fisher’s Exact test and a false discovery rate of 0.05.

### *In vitro* model

The 3D human *in vitro* triculture brain injury dataset analyzed in this study has been previously published (Liaudanskaya et al., 2023). In the present work, no new *in vitro* experiments were performed; instead, previously generated RNA-sequencing data were reanalyzed and incorporated exclusively for cross-platform comparison and evaluation of molecular convergence and scaling between human, *in vivo*, and *in vitro* models of cerebral injury. The original experimental methodology and primary outcome measures are described in detail in the prior publication (Liaudanskaya et al., 2023), whereas all integrative analyses, cross-platform comparisons, and interpretations presented here are novel to this study.

#### 3 D *in vitro* human triculture brain tissue model fabrication

3D brain-like scaffolds were generated using porous silk-collagen scaffolds coated with poly-ornithine and laminin, following our previously published protocols (Liaudanskaya et al., 2020, 2023; Sood et al., 2019; Tang-Schomer et al., 2014). The system incorporated human iNSC-derived neurons (Cairns et al., 2016), iPSC-derived microglia (ND41866C, Coriell; YZ1, UCSCC) differentiated using established methods (Abud et al., 2017; Liaudanskaya et al., 2023) and primary human astrocytes (Sciencell, cat. no. 1800). Neurons were maintained in KnockOut DMEM with GlutaMax and serum replacement, astrocytes in astrocyte growth medium with serum and growth factors, and microglia in DMEM/F12 with B27, N2, and cytokines (IL-34, MCSF, TGFβ).

When cultures reached near confluency, cells were collected, pelleted, and combined at a ratio of 2 million neurons, 0.5 million astrocytes, and 0.1 million microglia. Immediately before seeding, PLO/laminin-coated silk scaffolds were placed into 96-well plates, semi-dried, and seeded with 40 μL of the mixed cell suspension. After a 30-min incubation at 37 °C for attachment, 150 μL of neuronal medium (Neurobasal with 2% B-27, 1% GlutaMax, 1% Anti-Anti, and 1% astrocyte growth factors) was added. The next day, scaffolds were embedded in collagen type I (3 mg/mL, pH 7.0–7.2) and transferred to 48-well plates containing 1 mL of neuronal medium per well. Media was replenished every 4 days. Scaffolds were matured for 6 weeks, allowing stable neuronal networks and glial support to develop, before being subjected to controlled cortical impact injury.

For this study, we did not generate new scaffolds but instead reanalyzed RNA-sequencing datasets from the original study by the lead author, Liaudanskaya et al. (2023) Other results from that work (mitochondrial function, metabolomics, protein markers) were used only as supporting context to guide our hypothesis, but were not included in the re-analysis.

### 3 D model injury

#### Contusion injury [controlled cortical impact model (CCI)]

At 6 weeks of culture, the 3D brain-like scaffolds were transferred onto sterile weighing boats and subjected to a controlled cortical impact injury protocol. This was delivered using a pneumatic device fitted with a 3 mm flat tip, operated at 6 m/s with a displacement of 0.6 mm and a dwell period of 200 ms (Liaudanskaya et al., 2020). Control (sham) scaffolds underwent identical handling but were not impacted. Following either injury or sham procedures, scaffolds were maintained at 37 °C in a humidified incubator with 5% CO_2_ until they were collected 24 h after the injury. Downstream assessments included mRNA isolation for bulk RNA sequencing.

#### RNA isolation protocol

Scaffolds were collected 24 h after injury and immediately flash-frozen, and stored at −80 °C until RNA extraction. For isolation, samples were thawed on ice and mechanically fragmented into smaller pieces using scissors to maximize exposure to the lysis buffer. Each scaffold was incubated in 600 μL of RNeasy lysis buffer (Qiagen, Cat. No. 74106) for approximately 20 min to ensure thorough cell disruption. The lysates were further homogenized using QIAshredder Mini Spin columns (Qiagen) and centrifuged at 10,000 rpm to remove debris. The clarified supernatant was then combined with an equal volume of 70% ethanol (600 ml), mixed well, and applied in two sequential rounds to RNeasy Mini columns (Qiagen) at 10,000 rpm for 1 min each, allowing total RNA to bind to the silica membrane.

To eliminate genomic DNA contamination, on-column DNase digestion was performed using 30 μL of DNase I solution prepared in RDD buffer (Qiagen RNase-Free DNase Set, Cat. No. 79254), followed by a 15-min incubation at room temperature. Columns were then subjected to multiple washes with the RNA Wash buffer from Qiagen (Cat. No. 74106) to remove proteins, salts, and residual contaminants. After a final high-speed spin to ensure dryness of the membrane, RNA was eluted in 22 μL of nuclease-free buffer (Qiagen) by centrifugation at 12,000 rpm.

RNA concentration and purity were assessed using a NanoDrop 2000 spectrophotometer, with samples showing appropriate 260/280 absorbance ratios. The isolated RNA was stored at −80 °C until further use in sequencing workflows.

#### Messenger RNA (mRNA) sequencing and bioinformatic analysis

For transcriptomic profiling, approximately 1 μg of total RNA from each scaffold was subjected to quality control prior to library preparation and sequencing. RNA integrity was assessed using the Agilent 2100 Bioanalyzer, and all samples displayed high RNA integrity numbers (RIN), confirming minimal degradation. Libraries were prepared according to standard protocols and sequenced on an Illumina NovaSeq platform to generate 150 bp paired-end reads, with an average depth of ∼20 million reads per sample (Fiore et al., 2022a).

Raw sequence files (.fastq) were processed through the Tufts University Galaxy Server pipeline. Initial quality assessment was performed with FastQC^1^, and high-quality reads were aligned to the human reference genome (GRCh38.p13) using RNA STAR^2^. Read quantification was carried out with featureCounts, and differential expression analysis was conducted in R using the DESeq2 package, comparing injured and sham groups across experimental conditions (Fiore et al., 2022a,b; Liaudanskaya et al., 2023).

To interpret biological significance, multiple pathway-based and network-based approaches were applied. Gene set enrichment analysis (GSEA) was run using the Broad Institute desktop tool^3^, leveraging both the MitoCarta3.0 database and custom-curated mitochondrial gene sets. Co-expression network analyses were conducted using the CEMiTool R package, while additional GSEA utilized the Reactome pathway database^4^. High-confidence STRING interactions (score > 700) were incorporated for signed interaction network analysis, allowing integration of differential gene expression with protein–protein interaction data (Fiore et al., 2022a,b; Liaudanskaya et al., 2023).

#### Statistical analysis:

All statistical analyses were performed using GraphPad Prism version 9. Comparisons between two groups were made using two-tailed Student’s *t*-tests, while differences across multiple groups or conditions were assessed using either one-way or two-way analysis of variance (ANOVA), followed by Tukey’s *post-hoc* testing to identify specific group differences. A significance threshold of *p* < 0.05 was applied for all analyses. Each experimental paradigm was repeated independently a minimum of three times, with technical replicates ranging from two to six depending on the assay. Data are reported as mean ± standard error of the mean (SEM), and all graphical representations were generated directly in Prism.

### Translational analyses: integrating human, *in vivo*, and *in vitro* data

#### Statistical analyses for human data

Metabolites from the human metabolomic dataset were compared to target gene lists generated from *in vitro* and *in vivo* convergence. Any metabolites that were products of, targets of, or related to the genes of interest were selected, tested for across-season (i.e., Pre vs. Post) differences (Sign rank test, α = 0.05), and subsequently analyzed in regression and moderation analyses.

To assess if the identified metabolites had systems-level relationships, regression analyses were conducted using across-season changes in metabolite levels (△ = *Post* − *Pre*) as dependent variables (Y) and HAEs (cHAE_25G_, cHAE_80G_, aHAE_25G_, aHAE_80_G, and total number of sessions) and VR scores (Bal, RT, SM, and Comp) as independent variables (X). Cook’s distance was used for outlier removal and results achieving *p* < 0.05 were reported.

Permutation-based statistical moderation tests were conducted to investigate if Pre-season metabolite levels were moderated by end-of-season HAEs to affect (1) Post-season metabolite levels and/or (2) Post-season VR scores. Permutation-based methods were selected as they do not require (i) random selection of samples, (ii) sample independence, (iii) Gaussian distributions, (iv) large sample sizes for the central limit theorem, or (v) similar variance across samples. Instead, the only requirement is that the two samples being compared are interchangeable when setting up the null hypothesis (Fisher, 1935). Permutation tests re-sample observations from the dataset multiple times to build empirical estimates of the null distribution (Camargo et al., 2008). Permutation-based tests are especially applicable to studies with smaller sample sizes since they estimate statistical significance directly from the dataset rather than making assumptions about the underlying distribution. To perform permutation, the test statistic was obtained from the original dataset, the data were then randomly permuted multiple times (Q) and the test statistic was computed on each permutated dataset. The statistical significance was computed by counting the number of times the statistic value obtained from the permuted datasets was more extreme than the statistic value obtained in the original dataset (K), and then dividing that value by the number of random permutations (K/Q) (Camargo et al., 2008). Here, the following steps were followed where X is the independent variable (Pre-season metabolite), Y is the dependent variable (Post-season metabolite or Post-VR score), Mo is the moderator variable (HAE).

The moderation is characterized by the interaction term between X and Mo in the linear regression equation as given below:

*Y*=β_0_ + β_1_*X* + β_2_*Mo* + β_3_(X*Mo) + ϵ Moderation is significant if *p*β_3_≤0.05 and *p*_*F*_≤0.05, where *p*β_3_≤0.05 indicates that β_3_ is significantly different than zero using a *t*-test and indicates a significant interaction between X and Mo.

For this study, permutation-based moderation analysis was performed following the steps listed below and in Bari et al. (2021), Vike et al. (2022b):

1. Moderation analysis was performed by assigning the original data variables *x*_*i*_, *y*_*j*_, *z*_*k*_ as X, Y and Mo to obtain reference test-statistics: *t_0_* and *F_0_*.
2. Data permutation: values were randomly sampled without replacement from *x_i_* assign to $x_{i}^{\prime }$.
3. Similarly, $y_{j}^{\prime }$ and $z_{k}^{\prime }$ were computed.
4. Moderation analysis was performed on the permuted dataset $x_{i}^{\prime }$, $y_{j}^{\prime }$, $z_{k}^{\prime }$ by assigning as X, Y and Mo and the test statistics: $t_{q}^{\prime }$ and $F_{q}^{\prime }$ were obtained.
5. The counter variable *K_1_* was incremented by one if absolute value of $t_{q}^{\prime }$ was greater than absolute value of *t_0_.*
6. K_2_ was incremented by one if absolute value of ${{\text{F}}^{\prime }}_{\text{q}}$ was greater than absolute value of F_0_.
7. Steps 2–6 were repeated: *q* = 1,2, ⋯, *Q* times. Here, *Q* = 100,000.
8. Permutation-based *p*-value ${\text{p}}_{\beta_{3}}^{\text{perm}}$ was calculated as the proportion of the ${{\text{t}}^{\prime }}_{\text{q}}$ values that are as extreme or more extreme than t_0_ i.e., K_1_/Q.
9. Permutation-based *p*-value $p_{F}^{p\,e\,r\,m}$ was computed from *F_0_* and $F_{q}^{\prime }$ i.e., *K*_2_/*Q*.
10. Moderation analysis was considered significant if $p_{\beta_{3}}^{p\,e\,r\,m}\leq 0.05$ and $p_{F}^{p\,e\,r\,m}$ ≤ 0.05.

Significant permutation-based moderation results indicate that Pre-season metabolites (X) interact with HAEs (Mo) to model Post-season metabolite or Post-season VR scores (Y).

#### *In vivo* and *in vitro* data analyses–convergence of RNA sequencing datasets

*In vivo* and *in vitro* data RNA sequencing datasets were each independently analyzed as described above. Significant DEGs were identified within independent datasets, and convergent transcripts that were present and displayed significant differences in expression, regardless of the direction in both datasets, were identified and extracted manually using Excel. Convergent DEG’s were then sorted by directionality of expression differences elicited by independent injury paradigms. Convergent and parallel DEGs (i.e., DEGs present within both datasets altered in the same direction) were identified, and heat maps of data were generated using each independent injury paradigm’s respective controls. Using original expression data, each convergent and parallel DEG identified was converted to a percent control further to characterize convergence between *in vivo* and *in vitro* platforms. The Mouse Genome Informatics Gene Ontology Browser was utilized to identify DEG’s associated with metabolic processes that were convergent and parallel across both *in vivo* and *in vitro* datasets. The Gene Ontology enrichment analysis and visualization tool (GOrilla) (Eden et al., 2009) was used to identify cellular processes associated metabolically-related DEG’s present across *in vivo* and *in vitro* platforms. To generate correlation plots and statistically analyze convergence between models, DEG’s were analyzed using linear regression using Graphpad Prism for all plots, either solely convergent DEG’s or convergent and parallel DEG’s. GOrilla was used as an unbiased tool to identify pathways and subsequently isolate parallel and convergent DEG’s across platforms. These DEG’s were isolated and analyzed using linear regression in Graphpad Prism.

#### Transparency, rigor, and reproducibility summary

Sample sizes for all experiments were estimated using an α = 0.05 and power = 80%, in conjunction with means and standard deviation from pilot data and/or applicable published studies. Sample sizes are included where applicable within each respective experimental data figure. Outlier analyses (Grubbs’), where appropriate, were conducted with an alpha = 0.001 for all datasets obtained before statistical analyses. *RNAseq data from the human in vitro data* set was deposited to the GEO repository database^5^ with accession number GSE237013. RNAseq data from the *in vivo* data set is available, with corresponding subject metadata, within the supplemental data section provided. RNAseq data from murine *in vivo* data sets was initially analyzed using Illumina BaseSpace to identify deferentially expressed transcripts. All other data analysis was conducted using open-source software described herein. Murine subjects were obtained from commercial vendors and are readily available to the greater research community. All data will be made fully available to interested parties upon reasonable request. The data collected from athletes is available through Mendeley Data with DOI 10.17632/5tygm8sn24.1.

## Results

### Bedside-to-bench analysis

**FIGURE 2:**
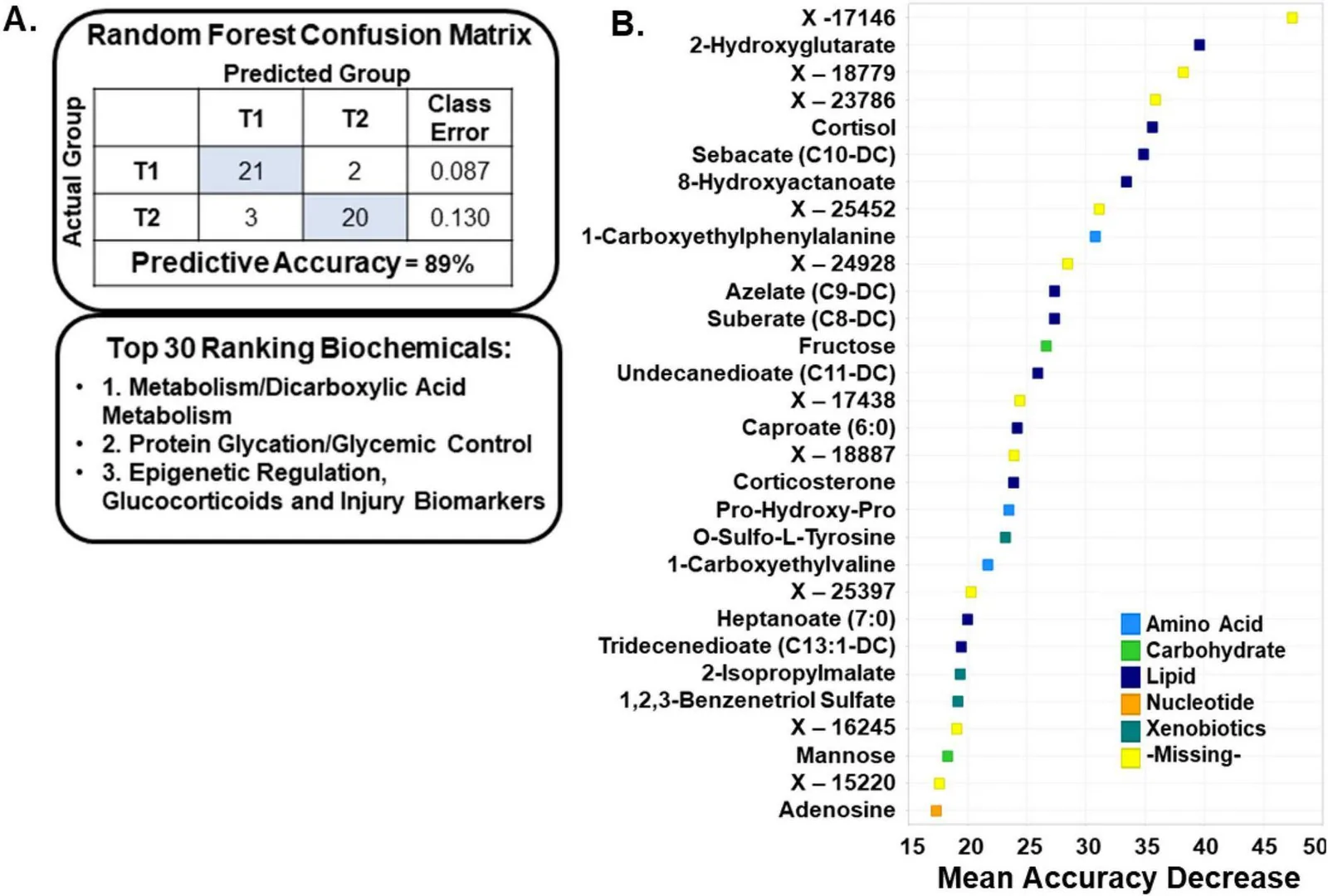
Random forest analysis and metabolite importance to discriminate Pre- from Post-season in collegiate American football athletes. **(A)** Metabolism/dicarboxylic acid metabolism; protein glycosylation and glycemic control; and epigenetic regulation, glucocorticoids, and injury biomarkers were key players in the random forest classification of human pre-season vs. post-season head impact biomarkers, achieving 89% predictive accuracy. *T1* was the first serum collection timepoint, occurring before any contact practices (pre-season). *T2* was the second collection timepoint, occurring within 1 week following the last regular season football game (post-season). **(B)** A biochemical importance plot highlights the 30 most important metabolites that distinguished pre-season from post-season timepoints in human athletes (Vike et al., 2022b). Importance was reported as the mean decrease in accuracy (MDA). Higher MDA values indicated that a given metabolite was more important to distinguish pre from post. “X-” designated unidentified metabolites at the time of analysis.

Blood samples were collected from, and serum metabolites were quantified for, American football athletes before (pre-season) and following (post-season) the competitive season [as described previously (Bari et al., 2021; Vike et al., 2022b)]. A statistical framework using random forest (RF) analysis was performed to identify feature importance via (i) testing the predictive power of pre-season metabolite levels with respect to post-season metabolite levels and (ii) identifying metabolites discriminated pre-season from post-season. From an initial bank of more than 3,000 metabolites, RF analysis revealed 30 metabolites that were most important to distinguish between pre- and post-season metabolomic profiles, with a predictive accuracy of 89% (Figure 2A). The feature importance analysis identified that changes in 2-HG, cortisol, and sebacate were the most important in characterizing the biochemical response to repetitive HAEs, and these three metabolites are directly connected to epigenetic regulation of cellular homeostasis. Collectively, the top 30 metabolites framed a hypothesis regarding the primary pathways contributing to pre- and post-season differentiation (Figure 2B): (i) dicarboxylic acid oxidation (e.g., sebacate); (ii) protein glycation and glycemic control; and (iii) epigenetic regulation, glucocorticoids and potential injury biomarkers (Abu Hamdeh et al., 2021; Berson et al., 2018; Chauhan, 2014; Cheng et al., 2023). Each of these functions are necessary for cellular homeostasis. This feature importance analysis identified 10 unknown metabolites as critical regulators of the biochemical response to HAEs, indicating the presence of (a) potentially unknown metabolic processes or (b) critical regulators of known pathways that have yet to be identified with metabolomics. This observation emphasizes the importance of identifying a convergent mechanism that could be interrogated to characterize important but unknown metabolites.

Utilizing inferences gained from ethological human data, we employed these data to drive the interrogation of molecular pathways altered by disparate forms of injury *in vivo* and *in vitro*, with the goal of refining convergent mechanisms across platforms that met criteria for scaling.

### Convergence between *in vivo* and *in vitro* TBI models

**FIGURE 3:**
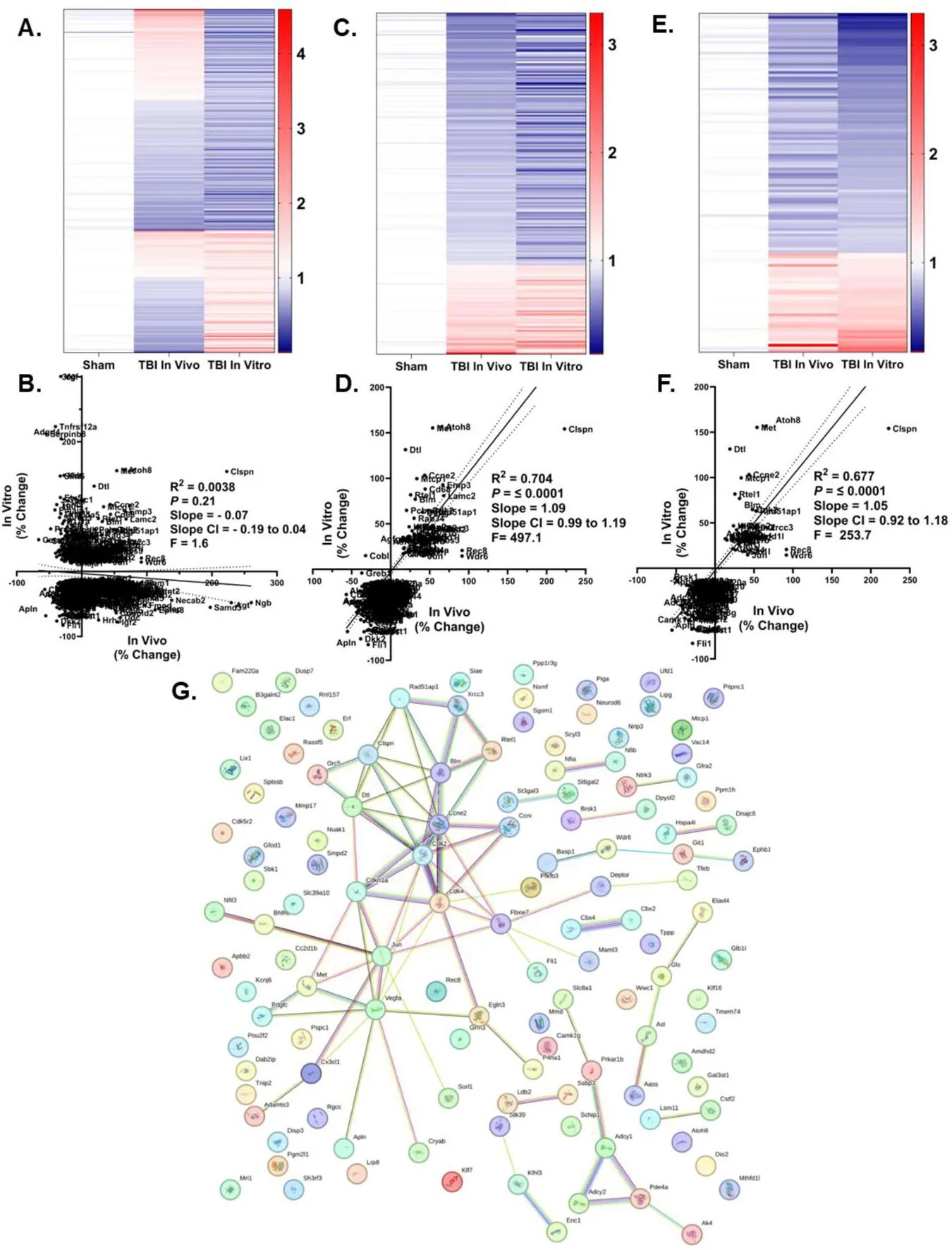
*In vivo* and *in vitro* RNA sequencing data sets aligned with human athlete metabolomes, demonstrating significant alterations of metabolism-related genes. For the *in vivo* model, HAEs were delivered to C57Bl/6 mice. For the *in vitro* model, 3D controlled cortical impact (CCI) was delivered to a human 3D triculture model. **(A)** Heat map of 411 differentially expressed mRNA transcripts 24 h after injury in *in vivo* impact models and human 3D *in vitro* contrasted with transcripts from an *in vivo* sham model. Gradient blue indicates a decrease, while gradient red indicates an increase [applies to panels **(C,E)**]. **(B)** Linear regression analysis of the differential mRNA transcript expression indicated no correlation between *in vivo* and *in vitro* RNA-sequencing data sets (R^2^ = 0.0038, *r* = –0.01337, *P* = 0.7870). **(C)** Heat map of the 211 differentially expressed genes were convergent (same direction of change) between *in vivo* and *in vitro* HAE injury models. **(D)** Linear regression analysis of the differential mRNA transcripts in panel **(C)** revealed a high level of correlation (R^2^ = 0.704, *r* = 0.8390, *P* < 0.0001) across the two impact models. **(E)** Heat map of the 123 convergent differentially expressed genes from panel **(C)** that are related to metabolism. **(F)** Linear regression analysis of the differential mRNA transcripts in panel **(E)** revealed a high level of correlation (R^2^ = 0.677, *r* = 0.8229, *P* < 0.0001) across the two impact models. **(G)** String plot analysis of the metabolic genes in panel **(E)** identified 34 hub genes that are involved in glycolysis, TCA, OXPHOS, and fatty acid oxidation, corresponding to processes predicted by human data (Figure 2).

Given a cellular homeostasis hypothesis in athletes, further transcriptomic analyses were performed in mouse *in vivo* (Logsdon et al., 2020; Reeder et al., 2022) and human *in vitro* (Dingle et al., 2020; Liaudanskaya et al., 2020; Snapper et al., 2023) models for injury. RNA sequencing data from the *in vivo* and *in vitro* models revealed 411 differentially expressed genes (DEGs), with 211 being altered in the same direction (Figure 3A). As expected, correlation analysis of all orthologous 411 DEGs from the *in vivo* and *in vitro* platforms, in the absence of pathway specification, revealed no statistically consistent relationship (Figure 3B; *P* = 0.21). In contrast, regression analysis of the 211 convergent and parallel gene candidates from both *in vivo* and *in vitro* platforms revealed an R^2^ value of 0.7 (Figures 3C, D; *P* < 0.0001), indicating that 70% of the variance was predicted by one platform in the other platform. When a salient majority of the information at one scale can predict information at another scale, the phenomenon of scaling is evident (Kim et al., 2010). Similarly, analysis of 123 DEGs specifically linked to cellular metabolic processes revealed an R^2^ value of 0.68 (Figures 3E, F; *P* < 0.0001), or 68% of the variance. Combined, these analyses establish a high level of specific convergence of transcriptional regulation between *in vivo* and *in vitro* platforms of two distinct injury models. From a biological standpoint, this argues for scaling between these two platforms (Kim et al., 2010) across a broad array of genes. This scaling represents a convergence across a range of RNA transcript expression from very minimal to strong, indicating that even subtle changes in gene expression were detected across both experimental platforms. This has implications for the molecular neuroscience community and promotes the consideration of all significant DEGs, not just those with the largest expression changes.

Given that 70% of the variance could be predicted from one platform to the other, we pursued a STRING plot analysis of convergent transcriptomic data between *in vivo* and *in vitro* domains (Figure 3G). This analysis shows a lack of association and/or protein-protein interactions between most of the transcript candidates and a singular cellular/metabolic process. No singular cellular/molecular process is responsible for, nor drives, the high level of convergence across the two distinct platforms and injury modalities. That is, there are several convergent, overlapping, altered cellular/metabolic pathways that contribute to injury regardless of trauma modality and/or model system, so the observation of scaling represents how information in one system predicts information in another system.

### Core convergent mechanisms

**FIGURE 4:**
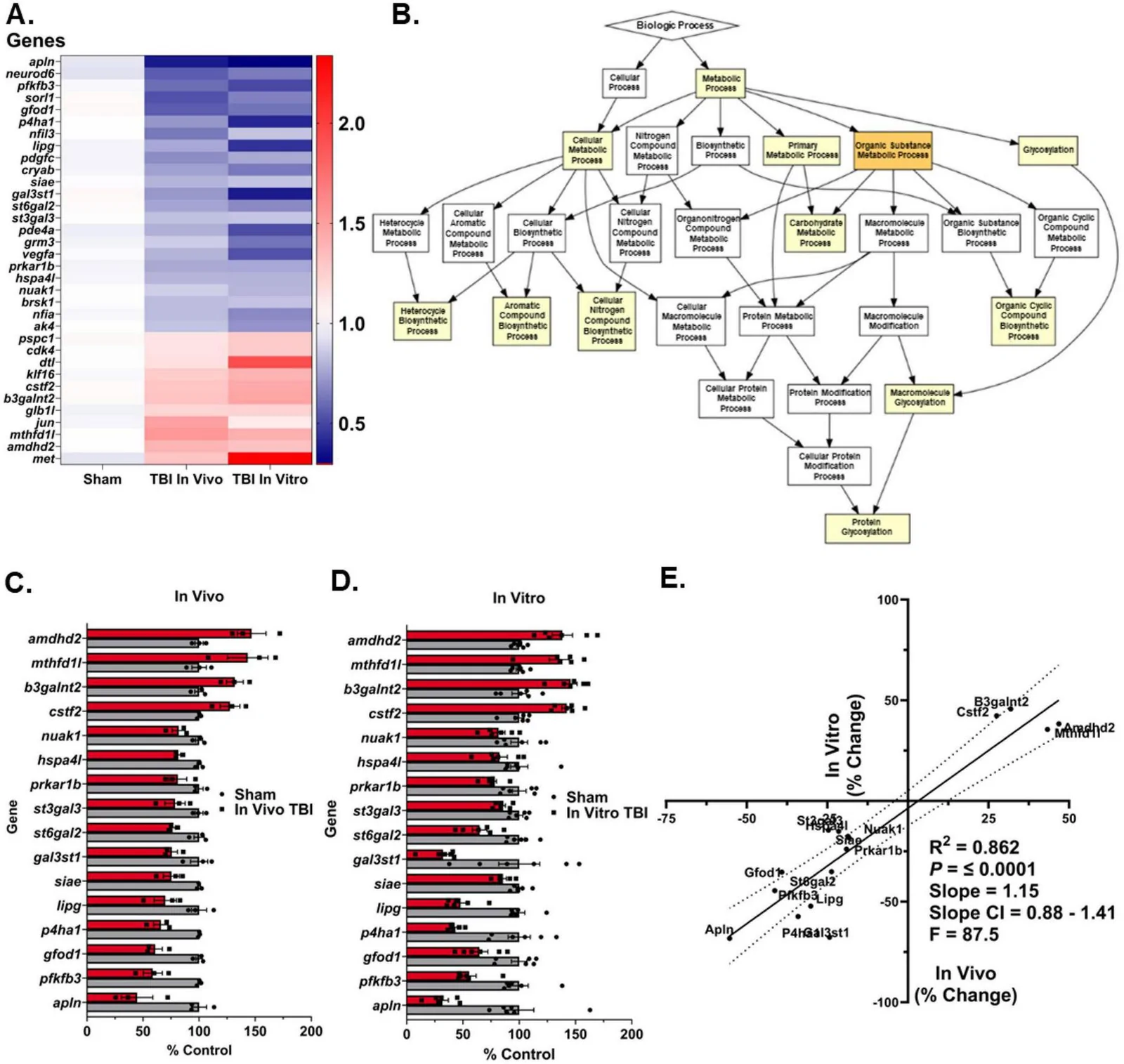
Hexosamine biosynthetic pathway, protein post-translational modifications, and epigenetic regulation are the core convergent mechanisms of injury-induced alterations in *in vivo* and *in vitro* models. **(A)** GOrilla pathway analysis of the 34 hub metabolic genes against the 411 differentially expressed genes (Figure 3A) predicts the involvement of protein O-, N- glycosylation. **(B)** Heat map of the 34 hub genes selected based on the string plot analysis in Figure 3G. **(C)** Mouse *in vivo* HAE impact model percentage change measures for the transcript expression of the 16 genes identified by the GOrilla pathway analysis in panel **(A)** associated with the Hexosamine biosynthetic pathway, protein O-, N- glycosylation, and sialylation from the 34 core metabolic transcripts in panel **(B)**. **(D)** 3D human *in vitro* controlled cortical impact (CCI) model percentage change measures for the transcript expression of the same 16 genes as in panel **(C)**. **(E)** Linear regression analysis revealed a very high level of correlation (R^2^ = 0.8621, *r* = 0.9285, *P* < 0.0001) for the changes in these 16 genes across the mouse *in vivo* **(C)** and 3D human *in vitro* **(D)** models.

The observation of scaling through high level convergence between *in vivo* and *in vitro* models implicated a number of metabolic pathways involved in cellular homeostasis. We further examined potential drivers of metabolic alterations, as summarized from the human data in Figure 2: (i) dicarboxylic acid oxidation, (ii) protein glycation, (iii) glycemic control, and (iv) epigenetic regulation induced by various injury modalities between *in vivo* and *in vitro* platforms. We used a combination of the GO-rilla visualization tool (Eden et al., 2009), Reactome, KEGG pathway analyses, and literature review to identify DEGs associated with metabolomic alterations in human athletes. These analyses identified 34 DEGs across the *in vivo* and *in vitro* platforms involving post-translational glycosylation (protein and micromolecular) and biosynthetic processes (heterocycle; aromatic, organic cycle, and cellular nitrogen compounds, and carbohydrates) (Figures 4A, B).

We further narrowed down a total of 16 differentially expressed transcripts (Figures 4C–E) linked specifically to sugar metabolism, the hexosamine biosynthetic pathway, and post-translational protein modifications through O- and N- glycosylation and sialylation pathways, including macromolecule glycosylation, protein glycosylation, and oxoacid metabolic processes. These processes were convergent with those identified with predictive metabolomic alterations observed in human athletes before and after exposure to repetitive HAEs, confirming the cellular homeostasis hypothesis observed in athletes, and validated with the *in vivo* and *in vitro* platforms.

Differential expression analysis of transcripts within *in vivo* and *in vitro* domains that were linked to macromolecule glycosylation and sialyation, similar to processes identified in human athletes, revealed an extraordinary level of relationship (R^2^ = 0.86, *P* < 0.0001; Figure 4E) despite the heterogeneity of injury modalities and model systems. In other words, information in the *in vitro* data predicted 86% of the information for the *in vivo* data, and vice versa.

### Bench-to-bedside analysis

Based on the identified convergence and scaling across three HAE models, including transcripts identified *in vivo* and *in vitro*, we examined whether disruption of cellular homeostasis following injury in athletes would drive changes in protein glycosylation through the manipulation of the hexosamine biosynthetic pathway (HBP), and its downstream effects on O- and N-linked glycosylation and sialylation. Such alterations in HBP and sialylation would be driven by injury-elicited metabolic dysregulation, which could result in functional consequences. To test this, an in-depth secondary analysis of 200 serum metabolites that were significantly altered pre- vs. post-season in human athletes was conducted to determine if any of the identified metabolites were linked to metabolic pathways downstream of classical carbohydrate metabolism, namely HBP, O- and N-linked glycosylation and/or sialylation networks.

**FIGURE 5:**
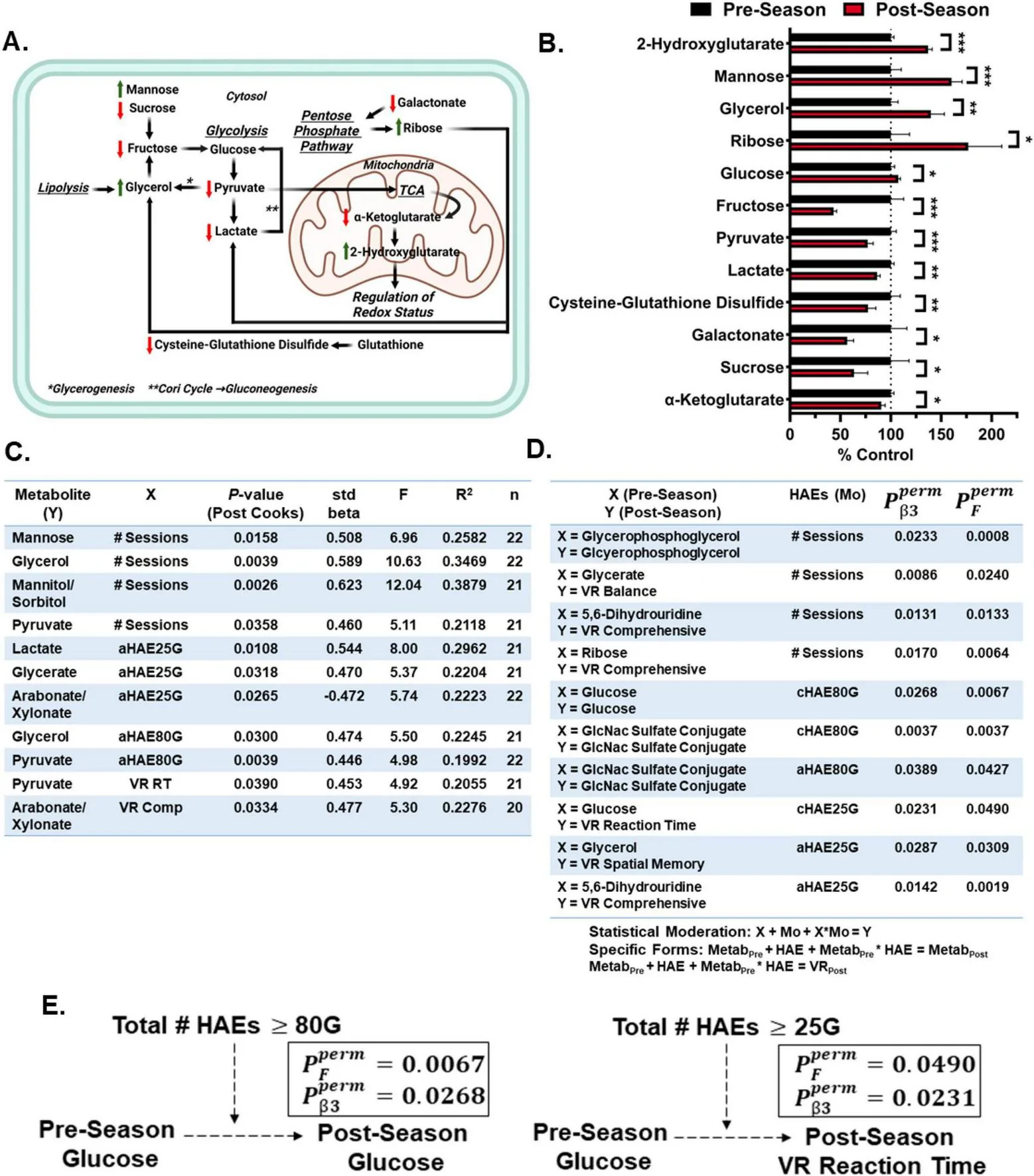
Analysis of human metabolomic data using convergent *in vivo* and *in vitro* gene list. **(A)** Twenty metabolites identified as convergent between human, *in vivo*, and *in vitro* models; 12 significantly differed between pre- and post-season (sign rank test, *p < 0.05, **p < 0.01, ***p < 0.001, respectively). **(B)** Bar graph plot demonstrating directional changes in the twelve metabolites from panel **(A)**. **(C)** Linear regression outcomes for changes in metabolites against post-season HAEs and across-season changes in VR scores (α = 0.05); includes standardized beta coefficients (std beta), F-statistics (F), R^2^ values, and sample size following outlier removal, *n*. **(D)** Permutation-based moderation framework analysis of metabolites [X = pre-season metabolite levels; Y = post-season metabolite levels or post-season VR scores; moderator (Mo) = HAE metrics]. Eight metabolites, all four VR scores, and all five HAE measures comprised 10 significant moderation relationships ($p_{\beta_{3}}^{p\,e\,r\,m}\leq 0.05$ and $p_{F}^{p\,e\,r\,m}$ ≤ 0.05). **(E)** Representative diagrams of two significant moderations. “Perm” indicates a permutation-derived *p*-value. # Sessions = total number of contact practice sessions, cHAE80G = cumulative number of HAEs over 80G; aHAE80G = average number of HAEs over 80G, cHAE25G = cumulative number of HAEs over 25G; aHAE25G = average number of HAEs over 25G; RT = reaction time; Comp = comprehensive score.

A subset of 20 serum metabolites was identified within this analysis that were linked to the TCA cycle, HBP, O- or N-linked glycosylation, and/or sialylation pathways identified (see Supplementary Table 1). Metabolite levels were altered from pre- to post-season and were related to HAEs and virtual reality (VR)-based measures of motor control and coordination in linear regression and moderation analyses (Figure 5). There were nine significant regressions with three different HAE measures (# Sessions, aHAE25G, aHAES80G) and two regressions with two VR scores (RT and Comp). In further moderation analysis, seven pre-season metabolites interacted with different HAE metrics to statistically predict post-season metabolite levels or VR behaviors (Figure 5). These findings revealed that the metabolite alterations are associated with functional (i.e., behavioral) consequences in collegiate football athletes, and scale within the human platform between molecular and behavioral measures.

Earlier studies in athletes identified not only metabolites as predictors of HAE-related behavior changes but also specific miRNAs (Chen et al., 2020; Vike et al., 2022b). We thus evaluated the miRNAs that were significantly affected post-season (Shi et al., 2009; Vike et al., 2022b) and assessed if these miRNAs were associated with the biosynthetic pathway through the miRDB database. This evaluation revealed that several genes involved in regulating HBP and sialylation are predicted to be regulated by miRNAs (e.g., miR-92a, miR-20a, miR-505, miR-195-5p) that were also altered in this same athletic cohort [see (Bari et al., 2021; Vike et al., 2022b)]. This evaluation found that miR-92a was predicted to regulate ST6GAL2; miR-20a to regulate SIAE and PFKFB3; miR-505 to regulate CSTF2 and B3GALNT2; and 195-5p to regulate HSPA4L, APLN, and CSTF2, which are all genes relevant to the biosynthetic pathway.

The metabolites and miRNAs [per (Vike et al., 2022b)] implicated in metabolic pathways identified through Figures 2–4 were significantly altered across the athletic season (Figure 5B) and play crucial roles in sugar metabolism, TCA processes, and epigenetics. Those linked with the TCA cycle (increased 2-hydroxyglutarate and decreased α-ketoglutarate) were altered in a manner consistent with reports of reduced energy metabolism (Shi et al., 2009), oxidative stress (Bartnik-Olson et al., 2013), hypoxia (Du and Hu, 2021), and epigenetic control of homeostasis (Abu Hamdeh et al., 2021; Berson et al., 2018; Cheng et al., 2023). The majority of sugar metabolites and pyruvate decreased, while mannose and glucose levels increased, suggesting a shift in glucose metabolism toward gluconeogenesis. Increased levels of glucose (Thomas et al., 2022) and mannose (Mondello et al., 2022) have been observed in acute TBI and are consistent with studies that report disturbed glucose metabolism, hyperglycolysis, and a resulting energy crisis stemming from attempts to meet post-TBI energy demands (Xu et al., 2020). Notably, these metabolites were either low in importance or not in the top 30 from the RF analysis, but still demonstrated significant relationships with HAEs and behavior. This suggests that even insignificant changes in these metabolites could induce functional alterations.

Changes in these metabolites were further assessed with linear regression to identify relationships with HAEs and behavioral measures of motor control and coordination (Bari et al., 2021; Vike et al., 2022b). These relationships show the congruency between these metabolites and (a) direct measures of injury and (b) collateral behavioral outcomes (Figure 5C). Several HAE metrics were linked with a larger change in sugars and metabolites involved in glycolysis (pyruvate) and oxidative phosphorylation (xylonate). The involved metabolites are established intermediates between glycolysis (a cytoplasmic process) and the TCA cycle (a mitochondrial process), indicating some level of dysfunction in energy processes between these two cellular systems.

**FIGURE 6:**
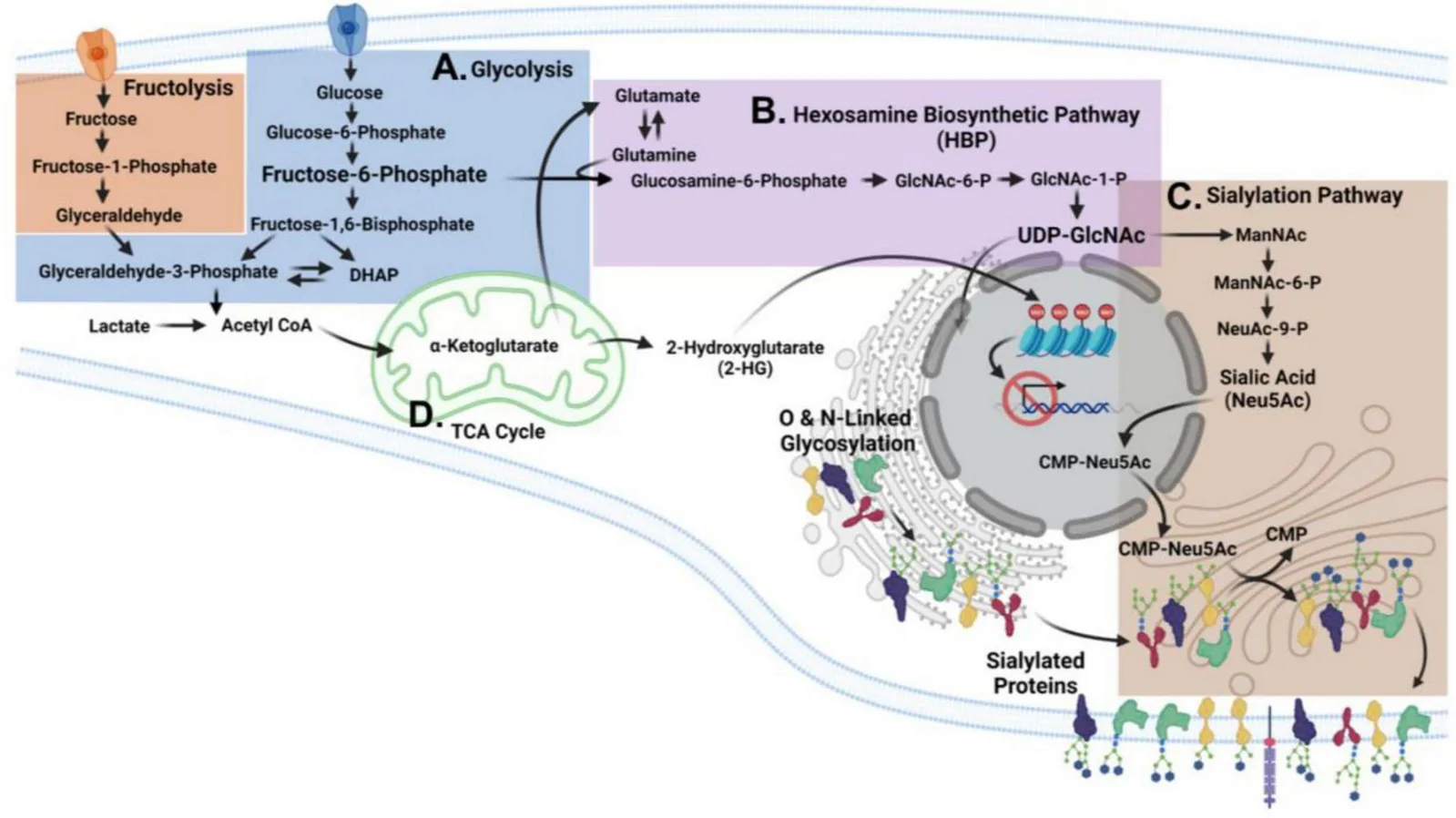
Different modalities of brain injury in human athletes, murine subjects and 3D *in vitro* cultures result in convergent shifts in metabolic homeostasis and transcriptional regulation involved in post-translational modifications and epigenetic regulation associated with neural information processing. Analysis of three disparate forms of brain injury indicated alterations in metabolic cascades associated with sugar metabolism and glycolysis **(A)** that compromise the hexosamine biosynthetic pathway (HBP) **(B)**, sialylation pathways **(C)** and the tricarboxylic acid (TCA) cycle **(D)**, the main producer of substrates and regulators of the epigenetic regulation and its downstream ATP production through oxidative phosphorylation.

As a final analysis of the statistical mechanism, moderation analyses were performed to assess if the interaction of pre-season metabolite measures and HAEs predicted post-season metabolite levels and behavior (Figures 5A–E). Eight metabolites, all four VR scores, and all five HAE measures were involved in 10 significant moderation relationships ($p_{\beta_{3}}^{p\,e\,r\,m}\leq 0.05$ and $p_{F}^{p\,e\,r\,m}$ ≤ 0.05). Two of these moderation relationships are displayed in Figure 5E, where HAEs (cHAE80G and cHAE25G) moderated the relationship between pre-season glucose levels and both (i) post-season glucose levels and (ii) post-season VR reaction time scores. That is, the interaction between pre-season glucose and HAEs drove the significant relationship between post-season glucose and post-season VR reaction time. This integration reflects scaling within platform and does not depend on significant changes across the athletic season but integrates them to predict a post-season measure. The targeted metabolites were involved in the HBP pathway and sugar metabolism, validating the cellular homeostasis hypothesis and highlighting this pathway as a target for further investigation (Figure 6). The results shown within Figures 5A–E further close the loop by confirming scaling within the human model for metabolites and pathways identified to scale across *in vivo* and *in vitro* platforms.

The observation of across-platform scaling for *in vitro* and *in vivo* data and within-platform scaling for human athlete data needs to be placed within a broader context of other studies showing scaling. Scale invariance has been reported for interval timing, where errors in time measures ramp up linearly with the estimated duration and relative errors depend on the subject or group but not the interval itself (Buhusi and Oprisan, 2013; Deisboeck and Kresh, 2006). For spatial measures, scaling is thought to exist not only along a single dimension but also across different levels of spatio-temporal organization within one organism or between different organisms. Humans and rats for instance, have opposite rest/activity cycles, whereby heartbeat fluctuations in both species exhibit similar power-law correlations over a range of time scales linked to the endogenous circadian rhythms (Hu et al., 2008). It should be noted that power-law correlations are a form of scaling - human judgment involving rewards and aversion assessments have been reported in multiple studies to follow power-law scaling from individual to group behavior (Kim et al., 2010; Strickland et al., 2017). The current results add significantly to this literature on scaling by demonstrating scaling across two platforms that can support future mechanistic study, and directly connect to findings of scaling within platform for humans, who reflect the actual clinical problem (i.e., animal and *in vitro* models are not humans with clinical problems despite what many publications seem to communicate).

## Discussion

For decades, the standard in neuroscience has been the combination of rodent *in vivo* and human ethological studies using post-mortem sources for data collection. Such a combination of data sources has brought outstanding discoveries in our understanding of circuitry and behavior (Pasko et al., 2022; Theofilas et al., 2014). The groundbreaking work by Takahashi et al. (2007) gave scientists induced pluripotent stem cells and, with that, human organoids that could spontaneously form brain-like tissue (Chiaradia and Lancaster, 2020; Lancaster et al., 2013) and other 3D brain-like *in vitro* models (Fiore et al., 2022a; Liaudanskaya et al., 2020, 2023; Snapper et al., 2023; Tang-Schomer et al., 2014), promoting further developments in our understanding of the cellular and genetic contributions to various diseases. None of these methods (*in vitro*, *in vivo*, or human ethology) have individually yielded definitive mechanisms that could be exploited to treat TBI or prevent the occurrence of serious TBI consequences. There is a need for a novel approach to identify and validate potential mechanisms that regulate the injury-, genetic- or environment-induced neurodegeneration. In response to this need, we integrated seemingly disparate human, *in vivo*, and *in vitro* TBI models to test for scaling of convergent pathways across platform and within platform.

This study observed consistent cellular metabolic cascades for TBI regardless of trauma modality (Figures 1–6). One model followed repetitive HAEs in collegiate athletes, the second utilized blast wave exposure leading to a singular HAE with loss of consciousness in rodents, and the third involved blunt impacts to a brain-like 3D cerebral cell culture model without any intervening skull, dura, pia, arachnoid, or vasculature (Figure 1). The observed consistency of molecular findings strongly supports the assertion that identification and subsequent targeting of convergent mechanisms underlying cerebral trauma and its associated comorbidities is possible through multimodal and multiscale platforms.

We used a closed-loop, multi-step analysis with the results obtained from each step informing the next step’s analysis until the analysis returned to the first dataset for final validation (Figures 1–6). At first, humans (metabolites and miRNA) demonstrated alterations to dicarboxylic acid oxidation, protein glycation, and glycemic control, epigenetic regulation, glucocorticoids, and potential injury biomarkers, all pointing to a hypothesis of disturbances in cellular homeostasis. We used these observations to fuel the mRNA analysis in both *in vivo* (mouse) and *in vitro* (human cell) models - systems differing in organizational complexity and trauma mechanisms. The summary of mRNA results indicated not only alterations to sugar metabolism and its downstream regulation of the vitally important O-, N- protein glycosylation, sialylation, and the hexosamine biosynthetic pathway (HBP), but also suggested significant changes to the epigenetic state through compromised sugar metabolism and its dependent TCA cycle (Figures 2–4). This data supported the cellular homeostasis hypothesis in athletes. Armed with a novel convergent mechanism between *in vivo* and *in vitro* models, with each platform predicting between 70% and 86% of the variance in the other and thus illustrating scaling across platforms, we went back and not only verified the involvement of HBP and alterations to post-translation protein glycosylation in the human dataset but additionally discovered a number of metabolites that were: (i) significantly related to HAEs and behavior (mannose, glycerol, mannitol, pyruvate, lactate, glycerate, and xylonate) and (ii) involved in moderation relationships with behavior and HAEs (glucose, glycerol-phospho-glycerol, glycerate, and glycerol, ribose, 5,6-dihydrouridine, and GlcNac sulfate) (Figure 5). This last analysis in the athletes demonstrated within platform scaling (e.g., between pre- and post-season molecular measures as moderated by HAE variables, and pre-season molecular measures and post-season behavior as moderated by HAE variables); this further validated the cellular homeostasis hypothesis by connecting miRNA, behavior, and HAE’s to metabolomics. The significant moderation effects in the athletes provide a framework for looking at scaling within platform for the same molecular measures found to scale across *in vivo* and *in vitro* platforms. Some of these metabolites did not show significant alterations between pre- and post-season, highlighting the importance of simple regression and statistical moderation to identify scaling effects and identify otherwise overlooked metabolic targets.

Across the three platforms studied, inherent differences exist that include tissue and structural complexity (gyrencephalic vs. non-gyrencephalic brain structure vs. cell culture), the presence of a blood-brain barrier and blood flow (human vs. mouse vs. cell culture), and identifiable discrepancies in cellular complexity (glial to neuronal cell ratio, myelination, and connectivity). Each platform also used a fundamentally distinct form of neurotrauma. The human model was not only ethological in nature, but none of the athletes were clinically diagnosed with a concussion during the investigation. Talavage et al. (2014) demonstrated that substantial changes in brain function occur without readily identifiable symptoms, and later work demonstrated significant differences in brain structure (Jang et al., 2019; Kashyap et al., 2022), function (Abbas et al., 2015; Breedlove et al., 2012, 2014; Fitzgerald et al., 2024; Robinson et al., 2015; Svaldi et al., 2017, 2020), and chemistry (Bari et al., 2019; Poole et al., 2014, 2015; Vike et al., 2022a,b) without any overt clinical symptoms. These changes were measured in 50%–95% of athletes (Nauman et al., 2015; Talavage et al., 2015), depending on the season and playing style of the teams participating in the studies. It should also be noted that surveys of college football players demonstrated nearly a six-fold greater incidence of concussions over the course of a season than was documented by attending physicians, suggesting that clinically observed symptoms are not commensurate with levels of neurotrauma (Baugh et al., 2015, 2020). Experimental and computational modeling (Liaudanskaya et al., 2020, 2023; Meaney and Smith, 2011; Snapper et al., 2023) results suggest that ethological HAEs can generate a wide range of peak accelerations, tissue strains, and strain gradients, depending on the impacting surface, location, direction, and even helmet type, suggesting that the injury patterns might be diffuse and athlete-specific (Meaney and Smith, 2011; Zhang et al., 2006).

This study built hypotheses based on human ethological data - metabolomics and miRNA from collegiate football players, where significant alterations were observed in sugar metabolism, TCA cycle, fatty acid oxidation (Vike et al., 2022b), protein glycation, and epigenetic regulators. Even with a random forest analysis, it is difficult to ascertain if these changes were due to repetitive cerebral impacts or high-intensity physical activity with or without associated orthopedic injuries. Parallel analysis of mouse *in vivo* and *in vitro* injury models were required to confirm if a common mechanism existed across the differing types of cerebral injury. *In vivo* studies used a blast and HAE injury model that generated a uniform, highly focused mechanical insult with strain fields that were highly localized in space and time (Logsdon et al., 2020; Reeder et al., 2022; Turner et al., 2013). The *in vitro* model utilized a rapid, localized impact on a three-dimensional tissue culture that included human cell-derived neurons, astrocytes, and microglia (Liaudanskaya et al., 2019, 2020, 2023; Snapper et al., 2023). While the macroscopic loading was compressive for the *in vitro* model, the porous three-dimensional scaffold experienced a complex array of compression, bending, and even tensile strains, all influencing load transmission to the cells residing in the embedded collagen gel. Consequently, all three trauma modalities imparted different forms of loading but managed to deliver traumatic injuries to neurons that are correlated through metabolic signatures. One limitation of this study is that the metabolic alterations predicted from the transcriptomic analyses in the *in vitro* and *in vivo* models were not directly validated by metabolomic profiling. Nevertheless, the convergence of independently generated human metabolomic data with transcriptomic changes identified in the mouse and human *in vitro* models supports the robustness of the identified pathways. Future studies integrating matched transcriptomic and metabolomic analyses across experimental models will be important to directly validate these predicted metabolic alterations.

In summary, this study explored whether shared biochemical cascades are conserved across diverse TBI platforms spanning humans, rodents, and human cell-based models. There is a widespread perspective that fundamental differences in injury paradigms result in divergent molecular and transcriptomic responses, particularly given the differences in cellular and tissue complexity. To address this question, we analyzed molecular disruptions across three distinct platforms (i) humans with repetitive HAEs, (ii) mice with singular blast-elicited HAE with loss of consciousness, and (iii) a human 3D *in vitro* model of blunt injury. Despite these differences, the results identified consistent alterations in three major metabolic pathways: (i) sugar metabolism, (ii) post-translational protein modifications, and (iii) epigenetic control of gene expression (Figure 6). The convergence of these pathways across diverse forms of brain injury suggests that fundamental metabolic responses to TBI are highly conserved, even following repetitive mild head impacts (HAEs) emphasizing that “nothing is mild about TBI at the cellular [and molecular] level[s] (Slobounov et al., 2014).”

## Data Availability

All data generated and analyzed during this study are available within this article and its Supplementary Information. The *in vitro* mRNA sequencing dataset generated for this study has been deposited in the NCBI Gene Expression Omnibus (GEO) repository (https://www.ncbi.nlm.nih.gov/geo/) under accession number GSE237013. The *in vivo* mRNA sequencing raw data are being deposited in the Open Data Commons for Traumatic Brain Injury (ODC-TBI), an NIH-supported repository for public data access. To ensure full accessibility of the data associated with the current manuscript, all applicable subject metadata, RNA sample information, and raw RNA sequencing data are also provided as a.csv file within the Supplementary Material.

## Footnotes

1
http://www.bioinformatics.babraham.ac.uk

2
https://www.ncbi.nlm.nih.gov/

3
https://www.gsea-msigdb.org/gsea/index.jsp

4
https://reactome.org/

5
https://www.ncbi.nlm.nih.gov/geo/
