# Supplementary material for: Molecular abnormalities scale across three models of cerebral injury
Source: Front Mol Neurosci. 2026 Aug 25;19:1844343. doi: 10.3389/fnmol.2026.1844343 (PMC13550177; doi:10.3389/fnmol.2026.1844343)
Supplement: Supplementary file 1 [file Data_Sheet_1.docx]

Supplemental Table 1. The list of 20 metabolites analyzed in Figure 5.

| **#** | **Metabolite** |
| --- | --- |
| 1 | mannose |
| 2 | a-KG |
| 3 | N-AG |
| 4 | GlcNAc SC |
| 5 | cysteine-glutathione disulfide |
| 6 | ribose |
| 7 | arabonate/xylonate |
| 8 | glycerol |
| 9 | glycerophosphoglycerol |
| 10 | pseudouridine |
| 11 | 5,6-dihydrouridine |
| 12 | 5-methyluridine (ribothymidine) |
| 13 | sucrose |
| 14 | fructose |
| 15 | mannitol/sorbitol |
| 16 | galactonate |
| 17 | glucose |
| 18 | pyruvate |
| 19 | lactate |
| 20 | glycerate |

Supplemental Table 2. Human athlete demographic information.

| **ID** | **Age** | **Race** | **YoE (yrs)** | **HoC (#)** |
| --- | --- | --- | --- | --- |
| 1 | 20 | White | 7 | 2 |
| 2 | 19 | African American | 6 | 0 |
| 3 | 21 | African American | 14 | 1 |
| 4 | 20 | African American | 9 | 0 |
| 5 | 19 | African American | - | 0 |
| 6 | 21 | African American | 9 | 0 |
| 7 | 21 | White | 12 | 0 |
| 8 | 20 | White | 12 | 1 |
| 9 | 21 | African American | 13 | 0 |
| 10 | 23 | White | 19 | 2 |
| 11 | 21 | African American | 11 | 0 |
| 12 | 19 | African American | 8 | 0 |
| 13 | 21 | White | 15 | 1 |
| 14 | 21 | White | 13 | 1 |
| 15 | 20 | White | - | 0 |
| 16 | 23 | White | - | 1 |
| 17 | 19 | African American | 13 | 1 |
| 18 | 20 | White | 7 | 0 |
| 19 | 23 | White | 14 | 1 |
| 20 | 21 | African American | 13 | 0 |
| 21 | 20 | White | 6 | 0 |
| 22 | 23 | African American | 8 | 0 |
| 23 | 22 | White | 13 | 0 |

YoE: years of experience playing American football

HoC: history of concussion where athletes provided the total number of concussions they had ever been diagnosed with (none occurred within the last year of play)

**Supplementary Figure 1:** Blast waveforms generated by murine model for blast– and acceleration-deceleration-induced traumatic brain injury (TBI). Model generates a blast wave that mimics the characteristics of a free-field Friedlander blast waveform, with a well-defined negative pressure phase, scaled in duration for murine subjects employing helium as a driver gas. Waveforms generated are reproducible, as outlined by 10 overlaid waveforms shown. Recordings were obtained using a PCB piezoelectric sensor precisely placed where the subject's head aligns during the experimental paradigm. The model causes mild injuries that result in acute increases in righting reflex times (RRTs), with no lethality, in murine subjects.

**Supplemental Figure 2:** Righting reflex times (RRTs) of cohort utilized for in vivo RNA sequencing studies following exposure to either sham or multimodal blast– and acceleration-deceleration-induced traumatic brain injury (TBI) paradigm. Subjects were exposed to either the sham or TBI experimental paradigm, and immediately following sham or injury treatments, the time to right themselves was recorded. Righting reflex times are typically utilized as a correlate of unconsciousness time in rodent subjects across various models for TBI. As depicted, the multimodal TBI paradigm results in a significant increase in RRT as compared to sham counterparts. Data analyzed in GraphPad Prism, using a Welch’s T test, P = 0.0273, t = 3.987, df = 3.052.
